# The undermining effect of facial attractiveness on brain responses to fairness in the Ultimatum Game: an ERP study

**DOI:** 10.3389/fnins.2015.00077

**Published:** 2015-03-10

**Authors:** Qingguo Ma, Yue Hu, Shushu Jiang, Liang Meng

**Affiliations:** ^1^Department of Management Science and Engineering, School of Management, Zhejiang UniversityHangzhou, China; ^2^Neuromanagement Lab, Zhejiang UniversityHangzhou, China

**Keywords:** facial attractiveness, Ultimatum Game, fairness, FRN, P300, decision neuroscience

## Abstract

To investigate the time course of the neural processing of facial attractiveness and its influence on fairness consideration during social interactions, event-related potentials (ERP) were recorded from 21 male subjects performing a two-person Ultimatum Game (UG). During this bargaining game, the male subjects played responders who decided whether to accept offers from female proposers, whose facial images (grouped as “attractive” and “unattractive”) were presented prior to the offer presentation. The behavioral data demonstrated that the acceptance ratio increased with the fairness level of the offers and, more importantly, the subjects were more likely to accept unfair offers when presented with the attractive-face condition compared with the unattractive-face condition. The reaction times (RTs) for five offers (1:9, 2:8, 3:7, 4:6, and 5:5) in the unattractive-face condition were not significantly different. In contrast, the subjects reacted slower to the attractive proposers' unfair offers and quicker to fair offers. The ERP analysis of the face presentation demonstrated a decreased early negativity (N2) and enhanced late positive potentials (LPPs) elicited by the attractive faces compared with the unattractive faces. In addition, the feedback-related negativity (FRN) in response to an offer presentation was not significantly different for the unfair (1:9 and 2:8) and fair (4:6 and 5:5) offers in the attractive-face condition. However, the unfair offers generated larger FRNs compared with the fair offers in the unattractive-face condition (consistent with prior studies). A similar effect was identified for P300. The present study demonstrated an undermining effect of proposer facial attractiveness on responder consideration of offer fairness during the UG.

## Introduction

Classic economic theory expects individuals to be rational and self-interest driven. For example, in a typical Ultimatum Game (UG), recipients were assumed to accept an amount of money to maximize gains. However, in a real bargaining situation, unsatisfying offers were often rejected by responders in favor of some notions of fairness. Empirical studies have demonstrated that proposers typically offered approximately 40% (fair offers) of the total money, and responders were more likely to reject 20% or less of unfair offers (Güth et al., 1982; Camerer, 2003; Sanfey et al., 2003; Oosterbeek et al., 2004). In addition to irrationality within humanity, several social factors influence individuals' fairness considerations, such as social distance (Campanhã et al., 2011; Wu et al., 2011a), initial ownership (Wu et al., 2012), self-contribution to income (Guo et al., 2014), social exclusion (Qu et al., 2013), and social comparison (Wu et al., 2011b, 2012).

Attractive faces can be perceived as informative or evolutionarily valuable in strategic games. Evolutionary theory states that facially attractive individuals are perceived to be physically healthier (Shackelford and Larsen, 1999), predict longevity (Henderson and Anglin, 2003), or have increased fertility (Thornhill and Gangestad, 1999). Zeng et al. (2012) performed a novel task of deciding whether to view an attractive female's picture or gain a certain amount of money while recording the male participants' brain activities. The results demonstrated stronger neural responses in the posterior regions related to the evaluation processes, which were induced by the attractive female pictures compared with the cash equivalents; these findings suggest that the attractive females were perceived as socially rewarding. An increasing body of evidence from functional Magnetic Resonance Imaging (fMRI) has also demonstrated that attractive faces activate the nucleus accumbens, orbitofrontal cortex and ventral striatum (Aharon et al., 2001; O'Doherty et al., 2003; Ishai, 2007; Winston et al., 2007; Cloutier et al., 2008; Tsukiura and Cabeza, 2011), which are all dedicated to the reward system, and thereby indicates a momentary equivalent role of facial attractiveness.

Our first concern in this study was how the brain responds to attractive faces. As we previously discussed, brain imaging studies have demonstrated that several brain regions are differentially responsive to attractive and unattractive faces (Aharon et al., 2001; O'Doherty et al., 2003). Evidence from electrophysiological experiments has illustrated the time course of the neural processes of facial attractiveness. An early posterior negativity (EPN) is more sensitive to attractive faces (Werheid et al., 2007) in the frontal brain region (N300, Zhang et al., 2011). Werheid et al. (2007) reported enhanced late positive potential (LPP) amplitudes in response to attractive compared with unattractive faces during an attractiveness classification task, which was consistent with previous findings (Johnston and Oliver-Rodriguez, 1997; Oliver-Rodriguez et al., 1999). A similar LPP effect was identified in other experiments (Schacht et al., 2008; Zhang et al., 2011). Chen et al. (2012) investigated the same two temporal stages of processing attractive and unattractive faces, including an early negativity (N2) and late LPP. However, the authors identified a contrary LPP pattern. Given the various findings regarding how facial attractiveness is processed, we continue to explore the temporal features of facial attractiveness processing using the high time resolutions of event-related potentials (ERPs).

In studies of labor markets, the theory of “beauty premium” and “plain penalty” has been noted by Hamermesh and Biddle (1993) in which attractive individuals were more likely to be hired and promoted, and workers of above average beauty earned approximately 10–15 percent more than workers of below average beauty. To date, several behavioral studies have investigated the effects of facial attractiveness on adherence to social norms, such as fairness (Solnick and Schweitzer, 1999) and cooperation (Mulford et al., 1998; Eckel and Wilson, 2006; Eckel and Petrie, 2011). However, the temporal dynamics of this process are rarely discussed. In this study, we investigated the influence of facial attractiveness on individuals' consideration of fairness during the UG. More specifically, we explored the “beauty premium” effect on fairness and its underlying neural mechanisms.

The UG is a stylized bargaining situation that has been widely used to study behaviors in social interactions. In this task, one player (proposer/allocator) proposes how to split a certain amount of money to another player (responder/recipient). The responder subsequently observes the offer and decides whether to object or accept it. If the offer is accepted, the money is divided as the proposer offered; otherwise, both individuals receive nothing. Several ERP publications have examined the brain responses to fair and unfair offers during the UG, which benefits from its high resolution time and is indexed by two ERP components, the feedback-related negativity (FRN) and P300 (Polezzi et al., 2008; Boksem and De Cremer, 2010; Hewig et al., 2011; Wu et al., 2011a, 2012; Alexopoulos et al., 2012; Luo et al., 2014).

FRN is a frontal-central negative deflection that peaks at 200–350 ms following the outcome presentation. ERP source localization and fMRI evidence suggest that the anterior cingulate cortex (ACC) is primarily responsible for negative feedback processing and FRN generation. According to reinforcement learning theory (RL-theory), the ACC activity is modulated by dopamine signals from the midbrain, where positive and negative prediction errors are coded. Negative prediction errors induced by unfavorable outcomes initiate phasic decreases in dopamine inputs and therefore result in increased ACC activity, which is reflected as a more negative FRN component (Gehring and Willoughby, 2002; Yeung and Sanfey, 2004; Ma et al., 2014). Studies regarding game theory and economic interactions have demonstrated that a violation of social norm could also generate an enhanced FRN response. For example, when responders encountered an unequal offer during an asset division, a more negative FRN was elicited compared with equal offers (Polezzi et al., 2008; Boksem and De Cremer, 2010; Hewig et al., 2011; Wu et al., 2011a).

Based on these studies, we expected a larger FRN for the unfair offers in our experiment. More importantly, this FRN pattern was predicted to be modulated by the attractiveness of proposers. Multiple researches (Campanhã et al., 2011; Wu et al., 2011a; Qu et al., 2013) have shown that allocator condition can differentiate individuals' brain responses to fair/unfair offers. For example, recipients became more sensitive to individuals who previously excluded them compared with an includer and a stranger and generated a more negative FRN in response to unfair offers (Qu et al., 2013). The modulation of social distance between recipients and responders on the recipients' fairness consideration was also discussed and found to influence both FRN and P300 components during the evaluation process of offers (Wu et al., 2011a). In this experiment, face images of proposers were classified into two groups based on level of attractiveness and presented to subjects who played as the recipients. As previous studies suggested that men are more sensitive and vulnerable to facial beauty (Buss, 1989; Cloutier et al., 2008; van Hooff et al., 2011), we recruited 24 male participants and presented them with 300 photographs of female allocators (half attractive, half unattractive), which enabled us to manipulate a one-shot UG across the experiment. Considering the reward equivalent function and theory of “beauty premium,” recipients' fairness toward attractive allocators may be attenuated and bring changes to FRN waveforms.

We also examined whether another ERP component, P300, would be influenced by the “beauty premium” effect. The P300 is the most positive peak in the 300–600 ms period after the presentation of feedback; it typically increases its magnitude from the frontal to parietal sites. Prior studies have demonstrated that P300 was sensitive to the magnitude and valence of reward and was more positive to a larger reward compared with a smaller reward (Yeung and Sanfey, 2004; Wu and Zhou, 2009), as well as to positive feedback compared with negative feedback (Hajcak et al., 2005, 2007; Wu and Zhou, 2009). In its extension to game theory, P300 was more positive during fair offers compared with unfair offers (Wu et al., 2011a, 2012; Qu et al., 2013). The similar pattern of P300 was expected to be observed in our experiment.

## Material and methods

### Participants

Twenty-four right-handed male subjects participated in this study. The participants were all students of Zhejiang University aged 18–26 years (*M* = 22.19 years, *SD* = 1.78 years). They were all native Chinese speakers with normal or corrected-to-normal vision and had no history of neurological disorders or mental disease. Informed consent was obtained from all subjects prior to the ERP experiment. The data from three subjects were discarded for excessive recoding artifacts and misunderstanding the rules of the bargaining task; thus, 21 valid subjects were included in the final data analysis. This experiment was approved by the Ethical Committee of Neuromanagement Lab of Zhejiang University.

### Experimental procedure

All facial images were obtained from the CAS-PEAL-R1 Face Database, photo pools of PKU and CAS and the Internet; the images were unfamiliar to the subjects (no movie stars, singers or other celebrities). All faces were gray processed by Photoshop software and edited to a uniform size (4.5 by 4 cm, 220 by 200 pixels). Three hundred images of Chinese female faces were rated for their attractiveness (from 1 = “not attractive at all” to 7 = “extremely attractive”) by 20 male students prior to the ERP experiment. The ratings of the two facial categories were compared with a paired *t*-test, and the attractiveness was significantly different [*M*_attractive_ = 5.13, *M*_unattractive_ = 2.07; *t*_(19)_ = 14.531, *p* < 0.001]. All 300 images were categorized into two groups: attractive-face and unattractive-face (each group had 150 female faces paired with 150 offers).

Another group of male subjects were seated comfortably in a dim, sound-attenuated, electrically shielded room. An introduction of the modified version of the UG was presented on written paper. The experimental stimuli were presented in the center of a computer screen at a distance of 100 cm. A keypad was provided to the subjects to make choices. The experiment consisted of three blocks, and each block contained 100 trials that featured 50 attractive and 50 unattractive facial images. Prior to the initiation of the formal experiment, each subject practiced 20 trials to familiarize themselves with the procedure.

**Figure 1:**
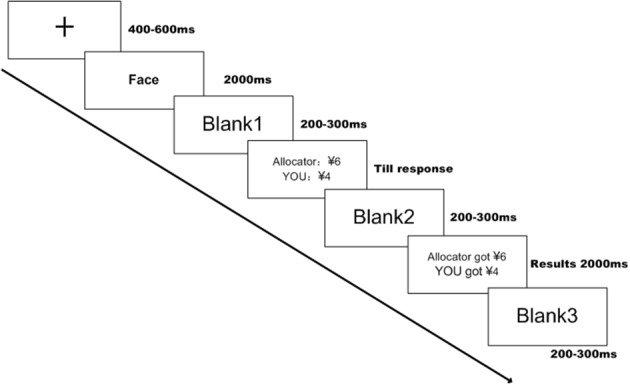
**A single trial of the experimental procedure**. The participants first saw either an attractive face or an unattractive face prior to the offer presentation. The participants made their choices by pressing the keypad with an unlimited time to make a decision. The screen subsequently displayed the final payoffs.

A single trial is illustrated in Figure 1. A fixation appeared at the beginning of each trial for 400–600 ms on a black screen. A photo of the allocator was subsequently presented for 2000 ms followed by her proposal of how to split ¥10 between herself and the responder. The subject (responder) had sufficient time to decide whether to accept or reject the offer by pressing the keypad. If the subject chose to accept the offer, then he and the female allocator would receive the amount of money she suggested; otherwise, they received nothing. Once he made the decision, the final income from this trial was presented on the screen for 2000 ms prior to continuing to another trial. The subjects were told that the photos and offers were collected in a previous experiment, and they were going to bargain with different female allocators who really existed. The payment for their participation was ¥30 (approximately $4.8) plus the income of two randomly selected trials. There were five different offer conditions (¥1, ¥2, ¥3, ¥4, and ¥5), and each offer was repeated 30 times. An average analysis was based on the trials that represented unfair offers (¥1 and ¥2) and fair offers (¥4 and ¥5). The counterbalance was manipulated among the subjects. The E-prime 2.0 software package (Psychology Software tools, Pittsburgh, PA, USA) was adopted for the stimuli presentation, triggers and response recording.

### EEG recordings

Scalp voltages were recorded (band-pass 0.05–70 Hz, sampling rate 500 Hz) with the Neuroscan Synamp2 Amplifier (Scan 4.3.1, Neurosoft Labs, Inc., VA, USA) using a 64-channel electro cap with Ag/AgCl electrodes according to the standard international 10–20 system. A cephalic (forehead) location was connected as the ground. The left mastoid was chosen as a reference, and the recorded EEGs were off-line re-referenced to the average of the left and right mastoids. An electro-oculogram (EOG) was recorded from electrodes placed 10 mm from the lateral canthi of both eyes (horizontal EOG) and above and below the left eye (vertical EOG). The electrode impedance was maintained below 5 kΩ during the recording.

### Data analysis

For the behavioral data analysis, repeated measures ANOVA was used to compare the acceptance ratios and reaction times (RTs) across 10 conditions: 2 (attractiveness: attractive and unattractive) × 5 (offer: 1:9, 2:8, 3:7, 4:6, 5:5). The *post-hoc* analyses were conducted using the Bonferroni correction.

The EEG data were analyzed using Neuroscan 4.3.1. The EOG artifacts were corrected followed by digital filtering through a zero phase shift (low pass at 30 Hz, 24 dB/octave). The EEGs were segmented for 1000 ms into epochs initiated at 200 ms before and 800 ms after the stimulus onset. The whole epoch was subsequently baseline-corrected by the 200 ms interval prior to stimulus onset. Trials that contained amplifier clipping, bursts of electromyography activity, or peak-to-peak deflections that exceeded ±80 μV were excluded from the final average. During the photo presentation, the EEG epochs were averaged for the attractive and unattractive faces. During the offer presentation, the EEG epochs were separately averaged for attractiveness (attractive/unattractive face) × valence (fair/unfair offer). Therefore, there were four conditions: attractive face-fair, attractive face-unfair, unattractive face-fair, and unattractive face-unfair.

Based on visual observations of the grand average waveforms and previous ERP studies (Wu et al., 2011b; Ullsperger et al., 2014) regarding outcome processing, two ERP components (frontal FRN and parietal P300) were analyzed. We averaged the ERP amplitude from the time range 270–340 ms and 350–550 ms post-offer presentation for the FRN and P300 analyses, respectively. According to the scalp distribution and previous reports, we selected nine electrode sites (F3, Fz, F4, FC3, FCz, FC4, C3, Cz, and C4) in the frontal and central areas for the FRN analysis and six electrode sites (CP3, CPz, CP4, P3, Pz, and P4) in the central-parietal areas for the P300 analysis. To investigate the effect across groups, a 2 (attractiveness: attractive and unattractive) × 2 (valence: fair and unfair offers) × 9 (electrodes) repeated measures ANOVA was conducted for FRN. A similar analysis was performed to examine the P300 amplitudes. A simple effect analysis was conducted when the interaction effect was significant. The Greenhouse-Geisser correction was applied in all statistical analyses when necessary.

To examine the neural processing of attractiveness of female allocators, the mean amplitudes of the frontal-central N2 (F3, Fz, F4, FC3, FCz, FC4, C3, Cz, and C4) and the central-parietal LPP (CP3, CPZ, CP4, P3, Pz, and P4) were further analyzed in the ranges of 240–280 and 350–550 ms post-onset of face presentation, respectively. Two-Way ANOVAs were conducted regarding the N2 and LPP components. The ANOVA factors were face (attractive and unattractive faces) and electrode sites. The degrees of freedom of the *F*-ratio were corrected according to the Greenhouse-Geisser method.

## Results

### Acceptance ratios

**Figure 2:**
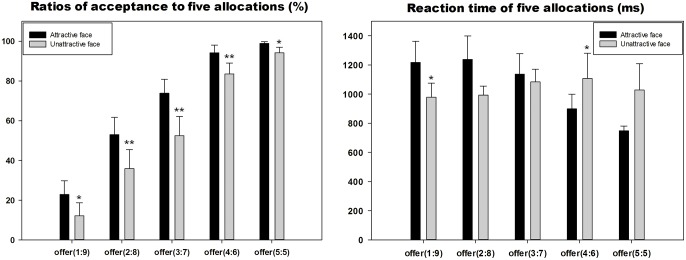
**The reaction time and acceptance ratio of five offers in two face conditions**. ^*^p < 0.05, ^**^p < 0.01.

The ANOVA analysis for the acceptance ratio (Figure 2) identified main effects of attractiveness [*F*_(1,20)_ = 16.709, *p* = 0.001] and offers [*F*_(1,20)_ = 43.893, *p* < 0.001]. The subjects accepted more offers in the attractive-face condition (mean ± SE, 68.6 ± 4.2%) compared with the unattractive-face condition (mean ± SE, 55.6 ± 5.5%). The acceptance ratio of offer (5:5) was significantly increased than offer (3:7) (*p* = 0.003), offer (2:8) (*p* < 0.001) and offer (1:9) (*p* < 0.001). However, a post-hoc comparison indicated there was no significant difference (*p* = 0.129) between offer (5:5) and offer (4:6). Offer (3:7) was accepted more than offer (2:8) (*p* = 0.002) and offer (1:9) (*p* < 0.001). A comparison between the two least fair offers was also significant (*p* = 0.022). The interaction effect was also significant [*F*_(1,20)_ = 3.426, *p* = 0.027]. All five offers were accepted more in the attractive-face conditions: for offer (1:9), *p* = 0.023; for offer (2:8), *p* = 0.009; for offer (3:7), *p* = 0.001; for offer (4:6), *p* = 0.007; for offer (5:5), *p* = 0.041. The acceptance ratios of each condition are reported in Table 1.

**Table 1:** **The acceptance ratio and reaction time during the offer presentations**.

	**Offer (1:9)**	**Offer (2:8)**	**Offer (3:7)**	**Offer (4:6)**	**Offer (5:5)**
**ATTRACTIVE-FACE**					
**Acceptance ratio (%)**	22.9 ± 6.9	53 ± 8.7	73.9 ± 7	94.2 ± 3.8	98.9 ± 0.8
**Reaction time (ms)**	1216.89 ± 145.52	1238.38 ± 160.38	1238.38 ± 160.38	898.67 ± 101.3	748.15 ± 32.64
**UNATTRACTIVE-FACE**					
**Acceptance ratio (%)**	12.2 ± 6.6	35.9 ± 9.6	52.5 ± 9.6	83.5 ± 5.5	94.2 ± 2.8
**Reaction time (ms)**	978.63 ± 96.1	994.04 ± 60.6	1084.32 ± 86.06	1107.23 ± 172.81	1028.39 ± 180.33

### Response time

The ANOVA analysis for the acceptance ratios (Figure 2) identified a main effect of offer [*F*_(1,20)_ = 3.636, *p* = 0.041]. The subjects typically responded the quickest to offer (5:5) (mean ± SE, 888.27 ± 99.31 ms), which was significantly faster than offer (2:8) (*p* = 0.007) and offer (3:7) (*p* = 0.001); however, the main effect of attractiveness was not significant [*F*_(1,20)_ = 0.128, *p* = 0.725]. The interaction effect exhibited a marginally significant difference [*F*_(1, 20)_ = 3.453, *p* = 0.072] with longer RTs for offer (1:9) (*p* = 0.043) and shorter times for offer (4:6) (*p* = 0.02) in the attractive-face condition compared with the unattractive-face condition. Other comparisons between the two face conditions produced statistical differences. In the unattractive-face condition, the RTs of the five offers were not significantly different (Table 1). However, in the attractive-face condition, the results indicated significant differences between the five offer conditions: offer (1:9) to offer (4:6), *p* = 0.001; offer (1:9) to offer (5:5), *p* = 0.002; offer(2:8) to offer (4:6), *p* < 0.001; offer (2:8) to offer (5:5), *p* = 0.003; offer (3:7) to offer (4:6), *p* < 0.001; and offer (3:7) to offer (5:5), *p* = 0.006.

### N2 and LPP

**Figure 3:**
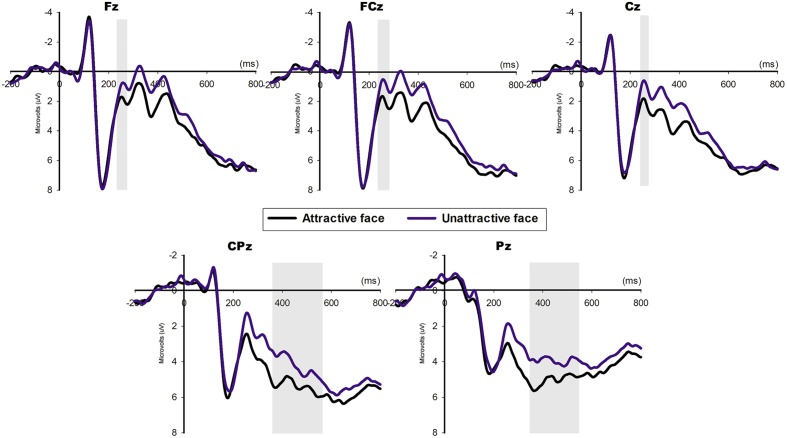
**The ERP grand-average waveforms and topographical maps of N2 and LPP at the midline Fz, FCz, Cz, CPz, and Pz for the attractive and unattractive face conditions**. The shaded 240–280 ms time window was used for the mean N2 amplitude. The shaded 350–550 ms time window at CPz and Pz was used for the mean LPP amplitude.

ANOVA analysis for N2 and parietal LPP (Figure 3) identified main effects of attractiveness with a more negative [*F*_(1, 20)_ = 8.253, *p* = 0.009] N2 elicited by the unattractive faces (mean ± SE, 0.954 ± 0.632 μV) compared with the attractive faces (mean ± SE, 1.875 ± 0.6 μV). A more positive [*F*_(1, 20)_ = 7.12, *p* = 0.015] LPP was elicited by the attractive faces (mean ± SE, 4.673 ± 0.618 μV) compared with the unattractive faces (mean ± SE, 3.581 ± 0.479 μV). The interaction between attractiveness and electrode for N2 was not significant [*F*_(1, 20)_ = 0.666, *p* = 0.612], but it was significant for LPP [*F*_(1, 20)_ = 6.209, *p* < 0.001].

### FRN

**Figure 4:**
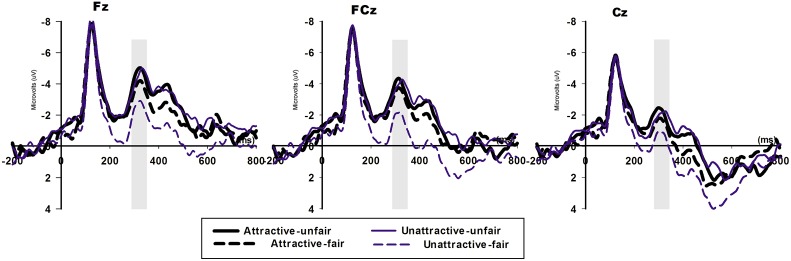
**The ERP grand-average waveforms of FRN at Fz, FCz, and Cz**. The time window for FRN was 270–340 ms.

As presented in Figure 4, the ANOVA analysis for the FRN identified a marginal main effect of attractiveness [*F*_(1, 20)_ = 4.299, *p* = 0.051] and a main effect of valence [*F*_(1, 20)_ = 11.073, *p* = 0.003]. Moreover, the interaction effect between attractiveness and valence was also significant [*F*_(1, 20)_ = 5.596, *p* = 0.028] with a more negative FRN for the unfair offers (mean ± SE, −2.576 ± 0.473 μV) compared with the fair offers (mean ± SE, −1.064 ± 0.529 μV) in the unattractive-face condition (*p* = 0.002) but not in the attractive-face condition (*p* = 0.115, unfair offers: −2.798 ± 0.608 μV, fair offers: −2.293 ± 0.636 μV).

### P300

**Figure 5:**
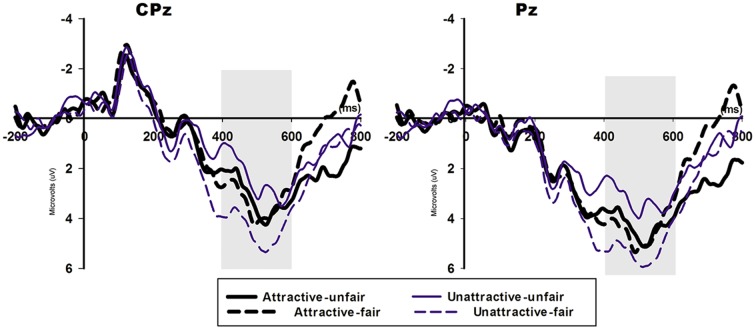
**The ERP grand-average waveforms of P300 for fair and unfair offers in the two face conditions at CPz and Pz**. The time window for LPP was 350–550 ms.

For the central-parietal component P300 (Figure 5), we identified a significant main effect for valence in which the fair offers (mean ± SE, 4.383 ± 0.689 μv) elicited a more positive [*F*_(1, 20)_ = 11.951, *p* = 0.002] P300 compared with the unfair offers (mean ±i SE, 3.153 ± 0.697 μV). A comparison between the two face conditions (attractive face: 3.731 ± 0.713 μV, unattractive face: 3.805 ± 0.685 μV) indicated no significant effect [*F*_(1, 20)_ = 0.853, *p* = 0.853]. The interaction effect between attractiveness and valence approached significance [*F*_(1, 20)_ = 3.956, *p* = 0.061], and the P300 was more positive for the fair offers (mean ± SE, 4.823 ± 0.724 μV) compared with the unfair offers (mean ± SE, 2.788 ± 0.758 μV) in the unattractive-face condition (*p* = 0.002) but not in the attractive-face condition (*p* = 0.413, unfair offers: 3.518 ± 0.768 μV, fair offers: 3.944 ± 0.745 μV). Additionally, the two face conditions produced similar P300 amplitudes in response to the unfair (*p* = 0.253) and fair (*p* = 0.099) offers.

## Discussion

The primary aim of this study was to elucidate how facial attractiveness modulated the response to unfair/fair offers during an UG. The behavioral data indicated stable RT results in the unattractive-face condition when the subjects responded to five offers, which suggests they followed a clearly defined strategy when bargaining with the unattractive-face group (similar to a previous study of fair and unfair offers Polezzi et al., 2008). However, a remarkable fluctuation in the RT was identified during the attractive-face condition, and the subjects delayed their decision making in the unfair conditions (1:9, 2:8, and 3:7) and responded quicker to the fair offers (4:6 and 5:5). More importantly, the subjects hesitated more when they received an offer (1:9) from an attractive allocator compared with when an unattractive female proposed an offer (4:6). Together with the findings of varied RTs in the attractive-face condition, this pattern implied that offers from proposers with increased physical attractiveness with mid-value offers, as presented in Polezzi et al.'s study (Polezzi et al., 2008), involved more complex decision making mechanisms with less predictable outcomes. The perceived attractiveness of the proposers could facilitate the subjects' reaction process toward a fair offer (4:6) and could also induce a struggle as to whether to accept an unfair offer (1:9).

A comparison of the acceptance ratio indicated that male recipients were more prone to accept an unfair offer if it was provided by an attractive female proposer. Solnick and Schweitzer (1999) first discussed the influence of physical appearance on decisions in an UG in which attractive females were often offered more than ordinary-looking individuals. The behavioral data in the current study were consistent with the notion of the “beauty premium.” (Campanhã et al., 2011) also reported a decreased rejection rate during the UG when participants bargained with a friend compared with a stranger. The authors attributed this effect to a fundamental bias caused by the friendship's effect on the subjective perception of fairness. In the present study, similar to the friendship effect, facial attractiveness can also be perceived as a type of subjective value (Cloutier et al., 2008; Zeng et al., 2012) that aroused reward-related neural circuitries (Aharon et al., 2001; O'Doherty et al., 2003; Senior, 2003). Furthermore, the questionnaires obtained after the experiment demonstrated that the subjects were more willing and looked forward to seeing the appearance of attractive faces. The impact of affections on the decision process during the UG has been demonstrated in multiple studies (Harlé and Sanfey, 2007; Andrade and Ariely, 2009; Kirk et al., 2011), and Andrade and Ariely (2009) pointed out the happy responder rejection rate (40%) of unfair offers during the UG was significantly lower than that of angry receivers (73%). Based on this evidence, the enjoyment of the perceived beauty could relieve the subjects' dissatisfaction with an unequal money distribution and attenuate their intention to decline unequal offers to punish the selfish proposers.

The ERP data first illustrated how the brain responds to facial attractiveness. A more positive LPP was generated between 350 and 550 ms in response to attractive faces compared with unattractive faces, which was consistent with previous findings (Johnston and Oliver-Rodriguez, 1997; Oliver-Rodriguez et al., 1999; Werheid et al., 2007; van Hooff et al., 2011; Zhang et al., 2011; Lu et al., 2014). LPP may reflect deliberative evaluation, and attractiveness could be perceived as a more emotionally significant stimulus, which would therefore enlarge the LPP amplitude. Additionally, considering the rewarding value of beauty (Aharon et al., 2001), attractive faces could draw more motivational attention from male subjects, which could evoke increased posterior positivity. An early negativity, N2, was also elicited from the frontal area to the parietal area, and it was larger in the unattractive-face condition. In a judgment task to categorize faces as “attractive” and “unattractive” (Zhang et al., 2011), an early negative component (250–350 ms) enhanced the response to unattractive faces. In a face recognition task (Grasso et al., 2009), mother generated a smaller N2 when perceived the picture of her son and much larger N2 when saw pictures of others, which was explained as a mismatch effect that mothers were always looking forward to seeing their children's faces and when they could not, cognitive control elicited an enhanced negativity. Similarly in our study, male subjects always anticipated to see beauty faces and when disfavored unattractive faces showed, elicited a more intense mental conflict and resulted in a more negative N2. Our findings further support the idea that the processing of facial attractiveness is initiated as early as 200 ms after the stimuli.

The FRN reflects the affective appraisal of negative events, such as a loss of money or a worse outcome than expected. An enlarged FRN in the frontal-central areas was identified when the participants received unfair offers (1:9 and 2:8) compared with fair offers (4:6 and 5:5), which is consistent with previous findings (Boksem and De Cremer, 2010; van der Veen and Sahibdin, 2011). The differential FRN responses to unfair and fair offers in the unattractive-face condition reflects the detection of a violation from the subjects' expectations (even asset distribution is an expected social norm) (Fehr and Gächter, 2002; Fehr and Fischbacher, 2004).

Surprisingly, we further analyzed the FRN in the attractive-face condition and demonstrated that this FRN effect disappeared. A similar pattern was observed in a dictator game (Wu et al., 2011a) when participants played with strangers or with one of their friends. Friendship was thought to be a factor that promoted an egalitarian distribution of assets; consequently, subjects' feelings and judgments of the strangers were weakened. In our experiment, the differentiated FRN patterns identified in the two face conditions suggested that male responders' sensitivity and dissatisfaction with inequality were stronger in the unattractive-face than in the attractive-face condition. Sanfey et al. (2003) demonstrated an activation of both emotional and cognitional-related brain regions when subjects performed an UG. Then involvement of emotional brain system in the UG was also supported by the evidences from participants' EEG, skin conductance responses (SCR) and subjective ratings of emotional valence to monetary offers (van't Wout et al., 2006; Hewig et al., 2011). Based on the correlation between mood change and FRN amplitude previously described (Qu et al., 2013), when subjects experiences a negative emotion in exclusion, they were subsequently more sensitive to the excluders' offers in the UG. Previous studies on social function of beauty showed that male subjects are willing to exert effort (Aharon et al., 2001) or sacrifice a certain amount of money (Eckel and Petrie, 2011; Zeng et al., 2012) to gain access to attractive female faces. The neural mechanism of the preference for beauty was outlined above that the perception of an attractive face could activate the reward circuitry of the brain and result in money-equivalent pleasure. Considering the rewarding effect of the beauty, when responders felt hurt by unequal treatment, the perceived attractiveness compensated for the negative emotions and distracted their fairness sensitivity. Therefore, we posited that it was subjects' enjoyment of facial attractiveness that weakened their consideration of fairness, decreased FRN responses for the comparative processes and resulted in the null effect of FRN.

Additionally, an increased P300 was identified in the fair condition in response to an increased amount of monetary allocation. In general, P300 is thought to represent the motivational significance (Yeung and Sanfey, 2004; Nieuwenhuis et al., 2005) and attentional allocation (Gray et al., 2004; Linden, 2005) of the outcome. The main effect of the valence of the offer with more positive responses to fair offers compared with unfair offers was consistent with previous studies (Wu et al., 2011a, 2012; Qu et al., 2013; Moser et al., 2014). Stronger P300 responses to fair offers indicated that the subjects attached more attention to larger momentary income and placed more value on equal divisions compared with unequal divisions.

In addition, a comparison of P300 for unfair and fair offers followed this general pattern in the unattractive-face condition; however, no valence effect was identified in the attractive-face condition. LPP, with a similar function in social evaluation as P300, was modulated by social comparison in an UG (Wu et al., 2011b; Moser et al., 2014). In Wu et al.'s study (2011b) social comparison was evoked when average level information was provided and highlighted to the subjects, and the subjects denoted more attention to the average comparison than their direct proposers. Due to this setting, the offer effect on LPP that moderately unfair offers elicited more positive-going responses compared with highly unfair offers only predominately appeared in an upward comparison when the subjects received more than average and vanished in other comparative conditions when the subjects were equally treated or received less. In a recent experimental setting, the offers not only included momentary meaning but also conveyed social information, such as the evaluation of perceived fairness and the proposers' looks, which could also evoke subject outcome comparisons with attractive or unattractive partners. Because the attractive-face condition also produced a lack of difference in P300 with respect to fair and unfair offers, this result can be explained as the offer effect on P300 was overshadowed by proposers' facial attractiveness that subjects attached no differential attentional resources to the two types of offers that originated from attractive proposers. In the unattractive-face condition, unfair offers were highly disfavored and captured less attention compared with fair offers, which therefore generated a smaller P300.

## Conclusion

To summarize, the “beauty premium” influenced responder fairness during the UG. Unfair offers from attractive female allocators were more acceptable to the male subjects, and the males presented with fluctuating RTs to the five offers in comparison with a stable reaction pattern in the unattractive-face condition. The ERP data supported the behavioral findings. In the early (FRN) and late (P300) stages of outcome evaluation, the subjects' fairness consideration was undermined by the “beauty premium,” which resulted in null FRN and P300 effects in the attractive-face condition. Additionally, the time course of brain responses to facial attractiveness illustrated that attractive faces elicited a decreased N2 and an enhanced LPP compared with unattractive faces.

### Conflict of interest statement

The authors declare that the research was conducted in the absence of any commercial or financial relationships that could be construed as a potential conflict of interest.
